# Preparation and Statistical Modeling of Solid Lipid Nanoparticles of Dimethyl Fumarate for Better Management of Multiple Sclerosis

**DOI:** 10.15171/apb.2018.027

**Published:** 2018-06-19

**Authors:** Smriti Ojha, Babita Kumar

**Affiliations:** ^1^Vishveshwarya Group of Institutions, Department of Pharmacy, G.B. Nagar, Uttar Pradesh 203207.; ^2^Sanskar Educational Group, Department of Pharmacy, Ghaziabad, Uttar Pradesh 201302.

**Keywords:** Box-behnken design, Dimethyl Fumarate, Multiple Sclerosis, Response Surface Method, Solid lipid nanoparticles, Polydispersity index

## Abstract

***Purpose:*** The objective of this study was to synthesize and statistically optimize dimethyl fumarate (DMF) loaded solid lipid nanoparticles (SLNs) for better management of multiple sclerosis (MS).

***Methods:*** SLNs were formulated by hot emulsion, ultrasonication method and optimized with response surface methodology (RSM). A three factor and three level box-behnken design was used to demonstrate the role of polynomial quadratic equation and contour plots in predicting the effect of independent variables on dependent responses that were particle size and % entrapment efficiency (%EE).

***Results:*** The results were analyzed by analysis of variance (ANOVA) to evaluate the significant differences between the independent variables. The optimized SLNs were characterized and found to have an average particle size of 300 nm, zeta potential value of -34.89 mv and polydispersity index value < 0.3. Entrapment efficiency was found to be 59% and drug loading was 15%. TEM microphotograph revealed spherical shape and no aggregation of nanoparticles. In-vitro drug release profile was an indicative of prolonged therapy. In-vivo pharmacokinetic data revealed that the relative bioavailability was enhanced in DMF loaded SLNs in Wistar rats.

***Conclusion:*** This study showed that the present formulation with improved characteristics can be a promising formulation with a longer half-life for the better management of MS.

## Introduction

MS is a demyelinating, autoimmune disorder and affects central nervous system.^1^ MS is a debilitating disease and accompanied by neurological symptoms of varying severity, which leads to accumulation of neurological disabilities over many years.^2^ The disease is mediated by a complex interaction of individual’s genetics and still unidentified environmental insults. In multiple regions the myelin sheaths deteriorate to scleroses, which are hardened scar or plaques.^3^ Dimethyl fumarate (DMF) is a fumarate derivative that is used as a dermatological agent in the treatment of psoriasis and skin disorder.^4^

DMF is recently approved by FDA for the management of relapsing-remitting multiple sclerosis and as per BCS classification it is a class 1 compound.^5,6^ In present practice DMF is being prescribed in two strengths of 120 to 240 mg as a delayed release hard gelatin capsule. DMF almost completely absorb from small intestine and is extensively metabolized by esterases before it reaches the systemic circulation. Compromised brain permeability, multiple dosing, poor patient compliance and economic hurdles are the other major challenges in proper utilization of DMF. The elimination half-life of DMF is approximately 1 hour. DMF is associated with the most prevalent side effect of abdominal pain, transient flushing, gastrointestinal irritation, erythema etc.^7^ Nanocarriers are an effective platform for a targeted delivery of hydrophilic and lipophilic drugs with increased stability.^8,9^ These approaches have some benefits such as increased drug stability, high drug payload, and potential colloidal therapeutic systems able to carry lipophilic or hydrophilic drugs or diagnostics, and no biotoxicity. SLNs are prepared by solid lipids and stabilized by surfactants, while NLCs are also prepared using solid and liquid lipids.^10,11^

Microemulsion based lipid nanoparticle preparation is simple, cost efficient, and also has considerable potential for acting as vehicles of drug delivery by incorporating a wide range of drug molecules.^12^ SLNs are a new generation of submicron-sized lipid emulsions where the liquid lipid (oil) has been substituted by a solid lipid. SLNs offer some unique properties such as small size, large surface area, high drug loading and the interaction of phases at the interfaces, and are attractive for their potential to improve performance of pharmaceuticals, neutraceuticals and other materials.^13^ The decrease in particle size is connected with a tremendous increase in surface area which is the responsible for the enhanced absorption and improved bioavailability of the drug. They have the potential target for prolonged drug release.^14^ The box behnken design is one of the most efficient designs of response surface experimental methodology to study the effect of formulation components on responses for exploring quadratic response surfaces and the second-order polynomial model.^15^ The box-behnken model is used to hit the target with reduced variability in experiments that increases the production yield and decreases the amount of waste, and represents opportunities for extensive financial gain.^16^

## Materials and Methods

### Materials

Components employed in the formulations in this research include stearic acid (m.p. 69.9°C), soy lecithin, tween 80, 1-butanol all are obtained from Chemsworth chemicals,Surat and DMF purchased from Alfa Aesar, a Johnson matthey company. Other chemicals were used only in analytical grade.

### Methods

#### Solubility study of DMF in lipid

The solubility study of DMF in lipid was based on previously described standard procedures.^17,18^ An excess amount of DMF was added to the lipids maintained at 70±5 °C were stirred thoroughly and sonicated for maximum solubilization. The solution was sampled at 2, 6, 8, 24, 48 and 72 h to analyze dissolved drug. Approximately 1 ml of the supernatant was transferred into a tared 10 ml volumetric flask and diluted with 7% v/v chloroform in methanol. Samples were further diluted prior to the analysis to allow quantification using the standard curve established. DMF concentrations were subsequently determined by UV-spectrophotometer. Equilibrium solubility was determined as the value when the solubility between two consecutive samples points does not differ practically.

#### Preparation of solid lipid nanoparticles

Microemulsion method developed by Gasco was used to develop Solid lipid nanoparticles with slight modifications.^19^ Solid lipid nanoparticles were prepared by diluting a warm emulsion (o/w) with cold water.^20,21^ Accurate quantity of stearic acid (as per the box-behnken design) was weighed and heated to 75°C until the entire lipid melts completely on water bath. To this lipid melt a co-lipid soy lecithin was added. DMF was dissolved in molten lipid mix at 75°C with stirring. Accurate quantity of surfactant (tween 80) was dissolved in distilled water. Temperature of aqueous phase was maintained at 75°C. The lipid phase was added to aqueous phase drop wise. After addition of each drop the aqueous phase was vortexed at 1200 rpm for 5 – 10 minutes and visualized for clarity of solution. If turbidity was observed the sample was sonicated for sufficient time at 75°C to obtain clear solution. The above clear dispersion was poured into distilled water kept at 2-5°C. The ratio of lipid microemulsion to cold water was kept as 1:20.

#### Box- behnken design for optimization of solid lipid nanoparticles

Design expert10^®^ software version 10.0.6.0 was used to develop and study the influence of three independent parameters namely lipid %w/w, surfactant %w/w and sonication time on two dependent variables namely particle size and drug entrapment efficiency. The independent factors and the dependent variables are listed in the Table 1.

**Table 1:** Level of variables in Box-behnken design

Independent variables	Low (-1)	Medium (0)	High (+1)
X1- Lipid amount (%g)	20	30	40
X2- Surfactant amount (%g)	2	4	6
X3- Sonication time (min)	1	2	3
Dependent variables	Constraints		
R1- Particle size (nm)	Minimum		
R2- %EE	Maximum		

The box-behnken three factors, three levels complete design consisted of 15 experimental runs with 3 central points and were performed in triplicate.

The design of experiment was applied to maximize the efficiency of experiments, to minimize number of experiments and to explore the quadratic response surfaces. The polynomial equation was generated by the experimental design is as follows^22^

Y= b_0_ + b_1_X_1_ + b_2_X_2_ + b_3_X_3_ + b_12_X_1_X_2_ + b_13_X_1_X_3_ +b_23_X_2_X_3_ + b_11_X_1_^2^+ b_22_X_2_^2^ + b_33_X_3_^3^

Where Y is the independent variable, b_0_ is the intercept and b_1_, b_2_, b_3_ are regression coefficients which was calculated from the experimental values of independent variables and dependent variables. Analysis of variance (ANOVA) identifies the significant independent factors which may affect the dependent factors and fitness of model.^23^All the batches of solid lipid nanoparticles were evaluated statistically (p < 0.05).

#### Average particle size

The average particle diameter, polydispersity index (PDI) and zeta potential value of the SLNs was determined by Photon correlation spectroscopy (PCS) DelsaNano C (Beckman Coulter Counter, USA) particle size analyser. The samples of SLNs were placed in disposable cuvettes for size and zeta potential measurement. The nanoparticles were dispersed in appropriate volume of HPLC grade water at 25°C, at detection angle of 90° for measuring size and PDI and 120° for zeta potential measurement.

#### Drug Entrapment Efficiency (% EE)

The % EE was determined as previously reported procedures.^24^ DMF loaded SLNs were separated from the solution by ultracentrifugation at12000 rpm for 1 hour. Supernatants recovered from centrifugation were decanted. DMF content in the supernatant was analyzed by a UV-Vis spectrophotometer at 208 nm. The percentage drug entrapment efficiency (%EE) was calculated using the formula give below^25,26^

[% EE= Total amount of DMF added-Free DMF in supernatant/Total amount of DMF added * 100]

#### In-Vitro Drug release study

*In vitro* drug release study of the optimized DMF loaded SLNs was carried out using the equilibrium dialysis technique at 37± 1 °C.^26-28^ Nanoparticles (equivalent to 1 mg DMF) were suspended in 5 mL of phosphate buffer (pH 7.4) and placed in a dialysis membrane bag. The membrane bag containing DMF loaded nanoparticle suspension was placed in 500 mL PBS and agitated at a speed of 50 rpm. Sink condition was maintained during the experiment and at regular time intervals, 5 mL of the aliquots were collected and replaced with an equal volume of fresh PBS. The collected aliquots were centrifuged at 12000 rpm and the supernatant was analyzed to calculate the cumulative % release of DMF using UV Visible Spectrophotometer at 208 nm.

#### FT-IR Spectroscopy

In order to evaluate chemical interaction between DMF and lipids spectra of the pure DMF, pure stearic acid and optimized SLN were obtained (by KBr Pellet Method) on FT-IR.^29^

#### X-ray diffraction

X-ray diffraction (XRD) study was performed to investigate the crystalline structure of SLNs, Stearic acid and the pure DMF.

#### Electron microscopic Examination

The optimized batch of SLNs was formulated and examined under transmission electron microscope (TEM) to study the morphology and degree of aggregation of prepared nanoparticles.

#### Stability Studies

Stability evaluation of optimized SLNs suspension was carried out to ascertain its future commercial viability. The SLNs were packed in screw capped amber colored glass bottles and was store at 2-8°C and ambient condition and at (28±4°C) for a period of 90 days. Samples were withdrawn at specified time intervals (1, 30, 60 and 90 days) and evaluated for the average particle size and residual drug content. The results of these parameters before and after the storage are compared and evaluated by means of ANOVA (p<0.05).

#### In-vivo pharmacokinetic study

The oral pharmacokinetic parameters were determined on Wistar rats of both the sex, weighing between 200 to 250 g and 5-6 weeks old. The protocol for animal study was approved by Institutional Animal Ethics Committee (IAEC) and Committee for the Purpose of control and supervision of experiments on animals. The animals were fed with a standard laboratory pellet diet and pure water *adlibitum*. The rats were divided into two groups each consisting of six animals. Group 1 was treated with pure DMF (50 mg/kg) and group 2 was treated with optimized SLNs formulations (equivalent to 3 mg/kg DMF). Blood samples (0.5ml) were collected from retro orbital plexus at predetermined time intervals and kept into heparin solution to prevent clotting. The serum was separated from blood samples by centrifugation. DMF rapidly metabolizes into its active metabolite mono methyl fumarate (MMF), and the content of both were determined using RP-HPLC.^30^

## Results

### Solubility study

The equilibrium solubility of DMF in Stearic acid was found to be 27 ± 3%w/v. This result predicts that stearic acid is capable to carry high drug load with minimum leakage. Thus stearic acid, a solid lipid could be a good candidate for formulation of SLNs.

### Model fitting and experimental design

A total 15 experiments with 3 central points, 3 levels and 3 factors were designed using three independent variables which were % of lipid, % of surfactant and sonication time in order to study their effect on two dependent responses which were average particle size and %EE. The replication of central point gives the result of experimental error.^31,32^ The experiments were designed using response surface modeling, box-behnken design with the help of Design Expert 10^®^ software. Except the three independent variables all other parameters were maintained constant. Total 15 formulations as mentioned in Table 2 of SLNs were formulated and analyzed for their response particle size and %EE. Response results of all the variables are listed in the Table 2.

**Table 2:** Response result of dependent variables

Runs	Factor 1	Factors 2	Factor 3	Response 1	Response 2
	**X1. Lipid % w/w**	**X2. Surfactant % w/w**	**X3. Sonication time (min)**	**Particle size (nm)**	**(% ) EE**
1	0	0	0	298	74
2	0	1	-1	322	88
3	-1	0	1	262	70
4	0	1	1	356	84
5	-1	0	-1	303	82
6	0	0	0	308	74
7	0	0	0	292	74
8	1	0	1	287	66
9	1	-1	0	298	76
10	0	-1	-1	356	75
11	0	-1	1	290	78
12	1	1	0	284	65
13	-1	1	0	300	87
14	-1	-1	0	291	63
15	1	0	-1	314	69

Fitting of model was done by sequential model sum of squares and model summary statistics. It was observed that within linear models, interactive models and quadratic models for particle size and for % EE, the quadratic model was found to be significant with p-value < 0.01.

The polynomial quadratic equation based on the analysis of Design Expert10^®^ was generated by the software and the quantification of the effect of independent variables on responses was done with the help of this equation.

Particle Size=299.33+3.37×A+3.37×B-12.50×c-5.75×AB+3.50×AC+25.00×BC-22.79×A2+16.71×B2+14.96×C2

% EE=74.00-3.25×A+4.00×B-2.00×c-8.75×AB+2.25×AC-1.75×BC-15.37×A2+4.13×B2+3.12×C2

**Figure 1:**
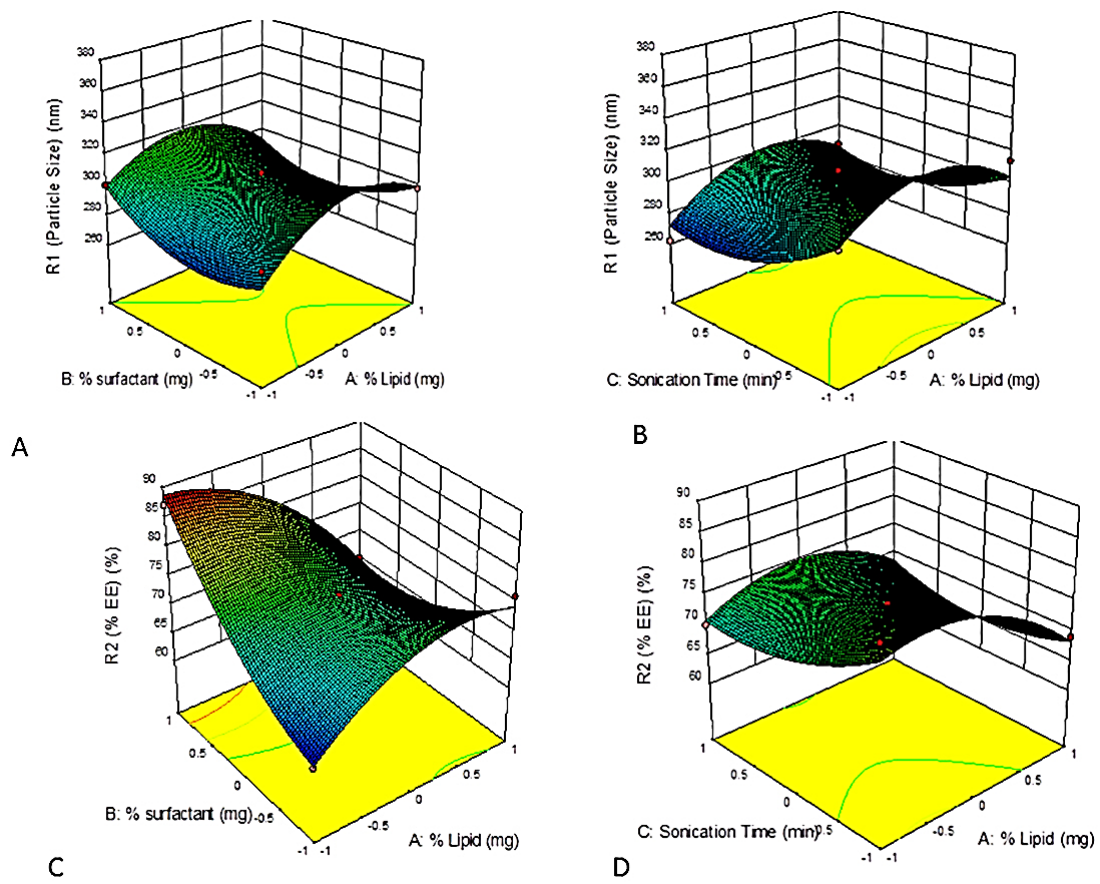
Response surface 3D plot for particle size (R1) and %EE (R2), A. Response surface 3D plot showing the effect of % lipid and % surfactant on R1, B- Response surface 3D plot showing the effect of % lipid and sonication time on R1, C. Response surface 3D plot showing the effect of % lipid and % surfactant on R2, D. Response surface 3D plot showing effect of sonication time and % lipid on R2.

The results were analyzed using ANOVA available in the software and the model was found to be significant with F value of 6.34 for particle size and 12.09 for %EE. The Response surface 3D graphs were constructed using the software. These graphs were used to study the interaction of independent variables on the responses, by keeping one of the variables at constant level. The Graphs obtained for the response particle size and %EE is depicted in the Figure 1.

### Numerical optimization 

Numerical optimization of SLNs was done by Design Expert10^®^ software and the response variables were optimized using RSM, box-behnken modeling. The optimized formulation was then prepared with 30% w/w stearic acid, 15% w/w surfactant and 4 min sonication time with desirability 0.87. The optimized batch of SLNs was further evaluated for average particle size, Zeta potential, PDI, % EE and % cumulative drug release. The observed responses of variables were compared with predicted responses and the calculated % error was found significant with values less than 0.05%.

### Size analysis and surface morphology

**Figure 2:**
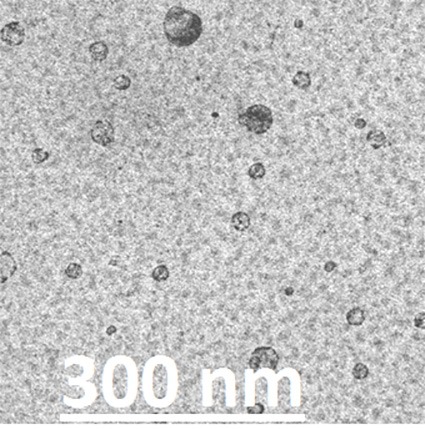
Transmission Electron Photomicrograph of optimized SLNs.

TEM microphotograph of optimized batch of SLN was obtained and shown in the Figure 2. The image revealed that the nanoparticles are of spherical shape and their average particle diameter was in the range of 300 nm. The average particle diameter as obtained by the Zetasizer instrument is 298 ± 4 nm (Figure 2), which also confirms the TEM result of particle size.

Zeta potential is the difference in the potential between the surface of tightly bound layer and the electro neutral region of the solution.^33^ The value of zeta potential of optimized SLNs was found to be -34.89 mv.

### IR spectroscopy and drug interaction

**Figure 3:**
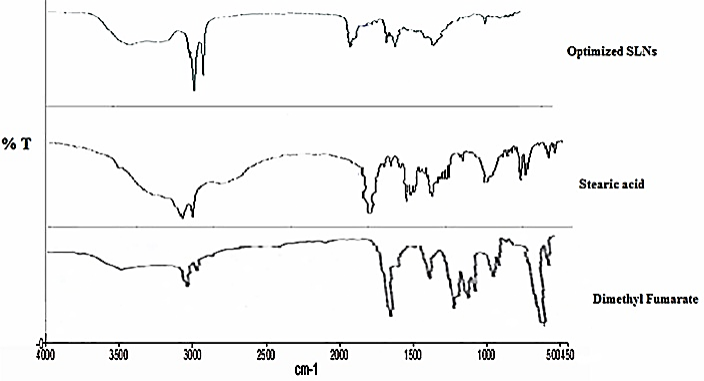
Overlay FT-IR spectra of DMF, stearic acid and optimized SLNs

IR spectroscopy method was used to study the drug–lipid interactions. IR spectroscopy of DMF shows all the characteristic peaks of functional groups present in the DMF. The absence of chemical interactions between drug and excipients were confirmed by the characteristic peaks of functional groups in the IR spectra of DMF, stearic acid and SLNs (Figure 3). The characteristic peaks of DMF were observed at 3428, 3022, 1719, 1445, 1297, 1164, 998, 889, 778 and 521 cm^−1^; for stearic acid 2900, 1450, 1467, 1385, 1395 cm^-1^. The characteristic peaks of DMF were also observed with IR spectra of SLNs which were at 3400, 2918, 2850, 1129, 1099 and 948cm^−1^. Similarly, the characteristic peaks of stearic acid were also observed at 2955 and 726 cm^−1^ in SLNs IR spectra.

### XRD analysis

**Figure 4:**
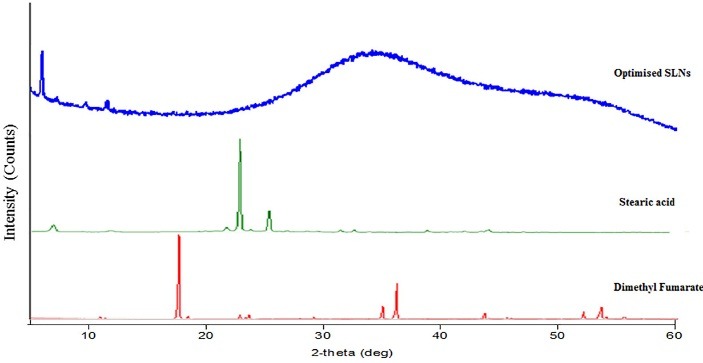
Overlay diffractograms of DMF, stearic acid and optimized SLNs

XRD studies were performed to indicate the reduction of the crystallinity of DMF in SLNs. The XRD of DMF, Stearic acid and optimized formulation were obtained (Figure 4). The diffraction spectrum of pure DMF and pure Stearic acid showed that the drug and the lipid were crystalline in nature. DMF presented many characteristic peaks observed in range 17.485, 20.013, 20.577and 30.47 at various 2θ values. The diffractograms of SA exhibit sharp peaks at 2h scattered angles of 7.07, 20.60, 21.71, and 24.05, indicating the lipid crystalline nature. Compared with the pure lipid, the peak intensities of SLN are much weaker, XRD pattern of SLNs showed that it is less crystalline and indicating amorphous nature of drug.

### In-vitro drug release

**Figure 5:**
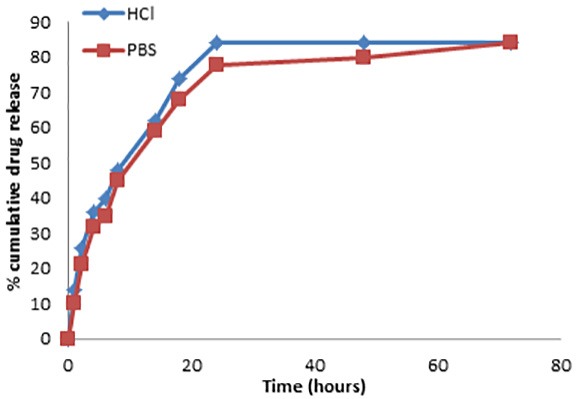
*In-vitro* cumulative % drug release profile of DMF from optimized SLNs.

*In-vitro* release profile of optimized SLNs was shown in the Figure 5. The cumulative percent drug release of DMF was 70.46±1.17% and 71.79±0.79 in 0.1 N HCl and 7.4 pH PBS respectively over a period of 24h. The percentage drug release of DMF was 52.24±0.85% in 0.1 N HCl and 54.55±0.45% in 7.4 PBS respectively after 2 h. Different kinetic models for *in-vitro* cumulative % drug release such as zero order (cumulative amount of drug released vs. time), first order (log cumulative % of drug remaining vs. time), Higuchi model (cumulative percentage of drug released vs. square root of time), korsmeyer-peppas model (log% cumulative release Vs. log time) and Hixson Crowell model (% drug remained Vs. time) were applied to study the drug release kinetics from the optimized formulations. Korsmeyer-peppas model was found to be the best‐fit model with a highest R^2^value of 0.903 in 0.1N HCl and 0.925 in 7.4 PBS.

### Stability study

The samples were analyzed for average particle size, % drug retained after 1 day, 30 days, 60 days and 90 days. Average particle size was found to be increased about 15 nm with storage at 28°C for 90 days, which was higher than the SLNs stored at 2-4°C. The result of % drug retained was found to be stable on storage for a period of 90 days.

### In-vivo pharmacokinetic study

The oral pharmacokinetic profile of pure DMF and DMF loaded SLNs is tabulated in Table 3. The pharmacokinetic parameters of pure DMF were compared with DMF loaded SLNs. In comparison with the pure DMF the values of C_max_, T_max_, and AUC were found to be increased with DMF loaded SLNs, which indicates the fast onset of action and long absorption phase of the present formulation. The half-life was also higher in SLNs formulation.

**Table 3:** Pharmacokinetic parameters

Parameters (unit)	DMF	DMF loaded SLNs
C_max_(ng.ml^-1^)	1054.51	1323.85
T_max_(h)	1.58	3.45
K_E_ (h^-1^)	0.187	0.165
K_a_ (h^-1^)	1.28	6.53
t_1/2_ (h)	3.5	5.8
[AUC]_0_^∞^ (ng.ml^-1^.h)	7398.84	18543.78
[AUMC]_0_^∞^ (ng.ml^-1^.h^2^)	94249.66	369762.97
MRT (h)	12.74	19.94

## Discussion

15 SLN formulations were prepared as per RSM box-behnken design by the method hot emulsification and ultrasonication. The model was fitted with help of software and quadratic model was found to be best fit model among all with predicted R^2^ value of 0.9853 and PRESS value of 9674 for particle size and R^2^ value of 0.9561 and PRESS value of 592 for %EE. The main focus of the design is to obtain maximum R^2^value and minimum PRESS value. The value of F< 0.05 indicates that the model terms are significant. A good correlation was observed between predicted and observed values with a higher value of R^2^.

Factors with positive values in the above quadratic equation indicated that the response increases with increase in factor value and negative value indicates an inverse relationship.^34,35^

It was clear by the graph that on increasing the amount of lipid the size of SLN also increases, which results into and increased surface tension.^36^ The decrease in average particle size was associated with an increased percentage of surfactant. This phenomenon is probably due to the hydrophobic interactions between the lipid and surfactant. It has been reported that the size of SLNs decreases as the ratio of phospholipids to stearic acid increases.^37^ Drug %EE increases as lipid percentage increases due to the high drug dissolution in an increased and molten lipid amount. Higher surfactant concentration resulted in lower entrapment efficiency, as this would increase the partitioning of drug from internal phase to external phase. This increased partitioning might result from increased solubility of drug in the external phase.^38^

The results of numerical optimization were used to formulate the optimized SLNs and evaluated for its particle size and % EE. Further analysis and statistical modeling revealed that the predicted responses are very close to the actual response results. A low value of prediction error validates the method and quadratic model.

Zeta potential measure is important to predict the long term kinetic stability of formulations as well as to understand the state of the surface morphology of the nanoparticles. This parameter is not only responsible for the stability of colloidal dispersions but it provides an indication of the degree of repulsion between particles with identical charge in the dispersion.^39^

Nanoparticles are considered to be kinetically stable with a zeta potential value higher than +25 mv or lower than -25 mv. Zeta potential analysis revealed that the optimized batch was stable with a zeta potential value of -34 mv. Negative value of zeta potential may be an indication of presence of fatty acid in SLNs preparation which provides rigid hydrophobic interactions. Higher zeta potential (either positive or negative) require higher energy for bringing two particles in contact with each other i.e. it possess high energy barrier in between the particles.^40^

TEM microphotograph of optimized batch of SLNs was obtained. TEM image clearly shows that the nanoparticles were spherical in shape with smooth surface morphology. The TEM study also revealed that the average particle size of SLNs were 300 nm. No particle aggregation was visible in the TEM microphotograph. As reported in various literatures it can be concluded that the lower size assures better brain delivery of the formulation which could be a good candidate for the treatment of MS.^41,42^

The XRD results seem to indicate that SA in SLNs was partially recrystallized or less ordered. These changes are because of the presence of a mixture of polymorphs in the nanoparticles and the method of obtaining the SLNs. It can also be seen that in the diffractograms of SLNs, the crystalline peaks of DMF is absent which could be because of conversion of crystalline DMF to amorphous DMF.

*In-vitro* release kinetics was fitted with various dissolution models and on the basis of correlation coefficient (R^2^) value the best fit model was selected. Korsmeyer-peppas model showed a highest R^2^ value of 0.903 in 0.1 N HCl and 0.925 in PBS with 7.4 pH indicated that release kinetics follows a diffusion coupled with lipoidal matrix relaxation. All the formulations exhibited an initial burst release followed by a controlled drug release for a period of 500 minutes. The initial burst release can be explained by the surface adsorption of DMF which is a main characteristic of nano and microparticles. Controlled release profile was observed which is mainly due to the diffusion of drug from inner lipid matrix.

The accelerated stability study data reveals that the prepared SLNs are stable and retains its property on storage. Further result showed a higher stability at low temperature conditions hence the prepared SLNs need to be stored at 5°C or low temperature to get a better shelf life.

The oral pharmacokinetic results confirmed that the bioavailability of DMF loaded SLNs was much higher as compared to free DMF which may be because of low particle size in SLNs. The increase in C_max_ and AUC values of SLNs indicates that the present optimized formulation may be helpful to reduce the dose of DMF. The relative bioavailability results of also confirmed that the present formulation may have an improved therapeutic activity and to reduce the dose of DMF. T_max_ value is delayed which can be explained by a sustained release rate profile of SLNs. MRT and half-life was also found to be increased which may be helpful to reduce the frequency of dosing and to reduce the side effects.

## Conclusion

DMF loaded SLN were successfully prepared by the method hot emulsion ultrasonication and optimized by three level three factors box-behnken design through RSM. The effect of all the three independent variables was studied and they are found to affect the dependent responses significantly. Observed response values were found to be in a linear correlation with predicted response values with a low predicted error result which also validates the quadratic model. The optimized formulation was prepared and characterized for particle size, % EE, zeta Potential and PDI. TEM image of the optimized SLN reveals a spherical shape and no aggregation of particles. FTIR study showed that the present SLNs formulation is free from any kind of drug interactions and the DMF original functional groups were present in the formulation also. The results of in-vitro study showed that the % cumulative drug release is 82% and approximately 40% of the drug was released in the first 180 minutes. Stability study was performed till 90 days and the results showed that the formulation is able to maintain its original property with insignificant changes. The results of the present study demonstrated that DMF loaded controlled release SLNs could be a promising candidate for the better management of the multiple sclerosis disease. Future in vivo study should be conducted to evaluate the effectiveness of the formulation in the management of the disease.

## Acknowledgments

The authors would like to thank the Vishveshwarya Group of Institution, G. B. Nagar for its support. The authors also extend this acknowledgment to CDRI, Lucknow for providing TEM images and FTIR spectra and IIT BHU, Varanasi for providing XRD diffractograms and results of zeta sizing.

## Ethical Issues

The study was approved by the Institutional Animal Ethical Committee and their guidelines were followed throughout the study.

## Conflict of Interest

All the authors declared that there is no conflict of interest.
